# The Role of the Ketogenic Diet in Lung Cancer: Current Evidence and Future Perspectives

**DOI:** 10.3390/cancers18081279

**Published:** 2026-04-17

**Authors:** Eleni D. Eleftheriadou, Serafeim-Chrysovalantis Kotoulas, Maria G. Grammatikopoulou, Anna Karakousi, Azoidou Maria, Aikaterini Trimpali, Xenofon Tsalampounis, Paschalis Evangelidis, Anastasios Vamvakis, Athanasia Pataka, Dionisios Spyratos

**Affiliations:** 1 2nd Pulmonary Department, General Hospital of Thessaloniki “G. Papanikolaou”, Leoforos Papanikolaou Municipality of Chortiatis, 57010 Thessaloniki, Greece; heleneleft@gmail.com; 2 Adult ICU, General Hospital of Thessaloniki “Ippokrateio”, Konstantinoupoleos 49, 54642 Thessaloniki, Greece; akiskotoulas@hotmail.com; 3 Immunonutrition Unit, Department of Rheumatology and Clinical Immunology, Faculty of Medicine, School of Health Sciences, University of Thessaly, Biopolis, 41223 Larissa, Greece; mgrammat@uth.gr; 4 Department of Nutrition and Dietetics Sciences, Hellenic Mediterranean University, 72300 Sitia, Greece; annoylakarakousi@gmail.com (A.K.); azoidoumaria2015@icloud.com (A.M.); katerinatrbl@gmail.com (A.T.); x.tsalampounis@gmail.com (X.T.); tvamvakis@yahoo.gr (A.V.); 5 2nd Propedeutic Department of Internal Medicine, General Hospital of Thessaloniki “Ippokrateio”, Aristotle University of Thessaloniki, Konstantinoupoleos 49, 54642 Thessaloniki, Greece; pascevan@auth.gr; 6 Respiratory Failure Clinic and Sleep Laboratory, General Hospital of Thessaloniki “G. Papanikolaou”, Aristotle’s University of Thessaloniki, Leoforos Papanikolaou Municipality of Chortiatis, 57010 Thessaloniki, Greece; patakath@yahoo.gr; 7 Pulmonary Department, Unit of Thoracic Malignancies Research, General Hospital of Thessaloniki “G. Papanikolaou”, Aristotle’s University of Thessaloniki, Leoforos Papanikolaou Municipality of Chortiatis, 57010 Thessaloniki, Greece

**Keywords:** ketogenic diet, lung cancer, cancer metabolism

## Abstract

**Background/Objectives**: Lung cancer (LC) remains the leading cause of cancer-related death worldwide, despite advances in systemic and targeted therapies. A mechanism of survival of tumor cells is metabolic reprogramming, characterized by increased glucose uptake, aerobic glycolysis, and alterations in mitochondrial function. These adaptations seem to support tumor growth, immune evasion, and therapeutic resistance. In parallel, supportive care and specifically nutritional interventions have become essential components of modern oncology. The interplay between metabolic reprogramming and targeted nutritional strategies represents a promising area of investigation that bridges tumor biology with supportive care, aiming to enhance both therapeutic efficacy and patient quality of life. **Methods:** This narrative review explores the biological and pathophysiological rationale for the ketogenic diet (KD) as a possible complementary intervention in LC management and summarizes the published preclinical and clinical data supporting this rationale. **Results**: We discuss key aspects of tumor metabolism, including the Warburg effect, glucose dependency, oxidative stress regulation, fatty acid metabolism, lactate cycling and tumor microenvironment interactions, with particular emphasis on how carbohydrate restriction and ketosis may exacerbate mitochondrial dysfunction in cancer cells and modulate inflammatory pathways. Furthermore, we summarize available preclinical and clinical evidence evaluating the KD in oncology and, more specifically, in LC, focusing on feasibility, safety, metabolic effects, and potential synergy with chemotherapy, radiotherapy, and immunotherapy. **Conclusions**: While preclinical models suggest enhanced treatment efficacy, clinical data remain limited and heterogeneous, with patient adherence representing a major challenge. Further well-designed longitudinal studies are required to clarify the therapeutic role of the ketogenic diet in lung cancer.

## Simple Summary

In this comprehensive review, the potential role of the ketogenic diet in lung cancer is examined across key biological and clinical domains, including cancer metabolism, current nutritional recommendations in oncology, mechanisms of ketogenic dietary interventions, and evidence from preclinical and clinical studies. Emphasis is placed on metabolic reprogramming in lung cancer, such as altered glucose utilization and mitochondrial dysfunction, and how ketogenic strategies may influence tumor growth, inflammation, and treatment response. Available data regarding the effects of ketogenic diets in other benign and malignant diseases are also discussed to provide a mechanistic context. Nutritional interventions have a place in lung cancer management, and even though the available data for the implementation of the ketogenic diet in humans are limited, there are sufficient mechanistic and preclinical data to support the need for well-designed clinical trials.

## 1. Introduction

Lung cancer (LC) is the most frequent type of cancer and is associated with the highest mortality rate, accounting for 20% of all cancer-related deaths worldwide [1]. Over the past decade, great achievements have been linked with LC treatment, especially with the introduction of immunotherapy across all disease stages, the deeper understanding of the molecular profile, and the increasing number of clinical trials focusing on new molecules as well as combinations of established therapies. Despite these advances, LC remains a major clinical challenge.

Palliative and supportive care represent an important aspect throughout cancer care. International guidelines state that these aspects should be supplemented early systemic cancer treatments, even from the time of diagnosis [2,3]. Within this context, cancer metabolism and its interaction with nutritional support remain important and promising areas for further study and analysis.

Cancer metabolism is a well-studied field; however, its use in clinical practice remains limited. During oncogenesis, cancer cells undergo metabolic reprogramming that enables them to meet the increased biological requirements in order to proliferate and survive [4]. These metabolic changes remain relatively common among the different cancer types, despite tissue heterogeneity, determining cancer behavior [5]. 

The first cancer-related metabolic alteration was described by Nobel laureate Otto Warburg in the 1920s, who observed that cancer cells consume large amounts of glucose and preferentially convert it to lactate even when oxygen is available [6]. Normally, healthy cells produce energy primarily through mitochondrial oxidative phosphorylation, in which pyruvate from glucose is fully oxidized to produce adenosine triphosphate (ATP), while under anaerobic conditions, pyruvate is converted by lactate dehydrogenase into lactate, through glycolysis, without additional ATP being generated at this step [7]. The “Warburg effect” is an example of metabolic reprogramming, which allows cancer cells to adjust their energy production in order to support their rapid growth and survival [4,6]. Other established mechanisms involved in oncogenesis are inflammation, oxidative stress, apoptosis, and overactivation of insulin and insulin-like growth factor-1 (IGF-1) signaling [8].

Dietary approaches that reduce glucose availability and alter energy metabolism, such as the ketogenic diet (KD), may influence these metabolic alterations of cancer cells and contribute as an additional management strategy against malignancies. This narrative review aims to summarize the basics of metabolic reprogramming, with a focus on LC, and to present the available preclinical and clinical data regarding the potential role of KD as an adjuvant strategy in LC management.

## 2. Materials and Methods

For this non-systematic narrative review, articles were retrieved from databases including PubMed, Web of Science, Google Scholar, and ClinicalTrials.gov. Searches were conducted using combinations of keywords such as “ketogenic diet,” “ketone bodies,” “Warburg effect,” “glucose metabolism,” “fatty acid oxidation,” “cancer,” “cancer metabolism,” “lung cancer,” “non-small cell lung cancer,” “tumor microenvironment,” “nutritional guidelines,” and combinations of these. A comprehensive literature search was conducted for studies published from January 2000 to December 2025, with earlier foundational studies included selectively providing historical context on cancer metabolism. Only articles published in English were included. The review considered a broad range of evidence, including preclinical studies, clinical trials, observational studies, case reports, and case series, as well as narrative and systematic reviews and selected abstracts from international conferences to capture the most recent developments. Reference lists of the identified publications were also screened to identify additional relevant studies. Studies were excluded if they lacked sufficient data to assess outcomes or were unrelated to the mechanistic pathways of the KD in cancer.

Studies were prioritized based on direct relevance to LC, study design quality, sample size, and the extent to which they provided mechanistic or clinical insights. Preclinical and clinical evidence are presented separately, with limitations of each clearly discussed. Preliminary conference abstracts were included; they were critically appraised by noting their lack of full peer review and the preliminary nature of their data. As this is a narrative review, no formal grading or meta-analysis was applied, and findings are presented qualitatively to provide a broad synthesis of the current landscape. Despite these measures, as a non-systematic narrative review, the possibility of selection and reporting bias cannot be fully excluded.

## 3. Discussion

### 3.1. Overview of Glucose Metabolism in Cancer Cells and the Possible Role of KD

**Figure 1:**
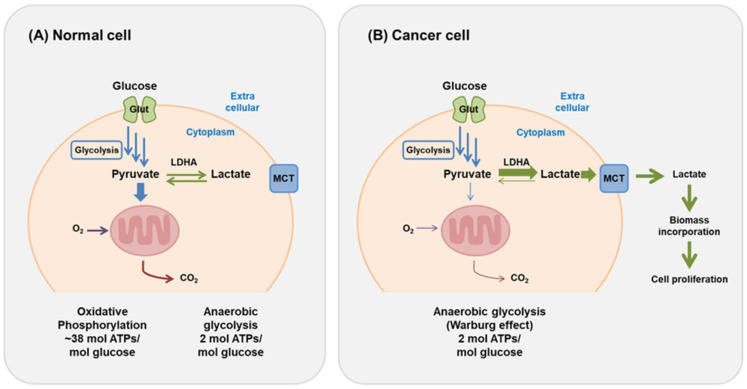
Overview of glucose metabolism in normal and cancer cells. (**A**) In normal cells, under normoxia, energy is produced by aerobic respiration, which includes glycolysis that takes place in the cytoplasm and pyruvate oxidation along with the citric acid cycle and the electron transport chain that takes place in the mitochondria. In this way, approximately 30–32 molecules of ATP are produced. Anaerobic metabolism, which occurs in the absence of oxygen, includes only glycolysis in the cytoplasm, with lactate being the product of the process. Glycolysis produces 2 ATP and 2 NADH per glucose molecule. In anaerobic conditions, pyruvate is transformed to lactate by lactate dehydrogenase, consuming NADH and regenerating NAD^+^ to sustain glycolytic flux. (**B**) illustrates the metabolic state of the cancer cell, in which anaerobic glycolysis is the main pathway for energy production. The Warburg effect demonstrates the preference of cancer cells for glycolysis even in normoxic environments. The lactate generated in the process of glycolysis is secreted and recycled via monocarboxylate transporters (MCTs), which play an important role in the energy metabolism of the cell and in the metabolism of lactate to provide carbon for biomass formation and cell growth and proliferation. GLUT, glucose transporter; LDHA, lactate dehydrogenase A; MCT, monocarboxylate transporter. Adapted from So-Hee Kim et al., 2021, licensed under CC BY 4.0 [9].

Aerobic respiration is the primary catabolic pathway for cellular energy production, in which glucose is fully oxidized to generate ATP, carbon dioxide, and water [7]. Anaerobic fermentation is a less efficient method of producing energy since it generates 16 times less ATP per molecule of glucose consumed compared to aerobic respiration. It seems paradoxical that the energy-demanding cancer cells rely on this pathway to survive, grow, and multiply (Figure 1) [7,9]. 

Several theories have been proposed to explain this phenomenon [6]. Anaerobic glycolysis is up to 100 times faster than oxidative phosphorylation; therefore, although fewer ATP molecules are generated, their rapid production can meet the increased energy demands of rapidly proliferating cancer cells while simultaneously regenerating NAD^+^ that re-enters the glycolytic pathway to sustain ATP production [9,10,11]. Additionally, elevated glucose uptake contributes carbon for the synthesis of cellular components [6,10]. In LC in particular, upregulation of glucose transporters (such as GLUT1) and glycolytic enzymes reflect an increased reliance on glucose uptake and anaerobic glycolysis (the Warburg effect) to sustain tumor growth and survival [11]. 

The possible anticancer effects of the KD are mostly based on the principle of altering the glucose metabolism in cancer cells. By reducing glucose availability—the primary energy source of cancer cells—KD shifts energy production toward mitochondrial oxidation, which, as discussed, is impaired in cancer cells. In this way, KD may reduce the availability of key nutrients for highly glycolytic tumor cells, which may compromise tumor cell survival. Accordingly, it contributes to the inhibition of the insulin/IGF-1 signaling pathway, which is a major factor in carcinogenesis. Many tumors overexpress glucose transporters such as GLUT1, indicating increased reliance on glucose metabolism [11,12]. It is also worth mentioning that hyperinsulinemia is related to a significantly higher risk of cancer mortality, as shown in a study including more than 9000 patients with cancer [13].

Beyond the Warburg effect, several additional metabolic pathways justify the use of KD in cancer therapy. NADPH and NADH are critical cofactors in cancer cell metabolism. A part of the consumed glucose is used in alternative metabolic pathways such as the pentose phosphate pathway (PPP), generating NADPH and pentoses. NADPH supports the biosynthesis of fatty acids, nucleotides, and neurotransmitters and protects cancer cells from oxidative stress by neutralizing reactive oxygen species (ROS), which are produced in excess due to mitochondrial dysfunction in cancer cells that would otherwise promote cellular damage and death [10,14]. On a KD, the PPP is downregulated due to reduced glucose availability, which limits nucleotide biosynthesis and antioxidant capacity [15].

KD also induces a metabolic shift from glycolysis toward oxidative phosphorylation (OXPHOS), increasing reliance on fatty acid oxidation (FAO) and ketone body metabolism. FAO provides acetyl-CoA for the tricarboxylic acid (TCA) cycle and generates reducing equivalents (NADH, FADH_2_) for ATP production. In some tumors, FAO may support survival by fueling OXPHOS and contributing indirectly to NADPH regeneration through linked metabolic pathways [16].

Non-small cell lung cancer (NSCLC) cells exhibit significant metabolic plasticity, frequently relying on fatty acids as an alternative energy source under nutrient-limited conditions. This metabolic adaptation is achieved both through mitochondrial FAO and increased lipid uptake from the tumor microenvironment (TME) via the overexpression of lipid transporters. [17]. However, this metabolic shift may also create vulnerability. In certain contexts, increased lipid utilization can lead to excessive lipid peroxidation, overwhelming antioxidant defenses and triggering ferroptosis and cell death [18]. Therefore, the impact of KD in NSCLC may depend on the balance between a tumor’s capacity to utilize fatty acids for energy and its susceptibility to oxidative stress.

Other cancer-surviving mechanisms are linked to factors that influence the TME. Vanhove et al. emphasize the heterogeneity of the TME of the LC. The ineffective tumor vascularization leads to different gradients of oxygen and nutrients within the tumor mass. Lactate cycling has been observed between oxygenated and hypoxic cells. Normoxic lung cancer cells utilize monocarboxylate transporter 1 (MCT1) in order to extract lactate from the TME and convert it into pyruvate for further oxidation, preserving glucose for usage by hypoxic cells [19]. On the contrary, low oxygenation induces other regulatory factors, such as the Hypoxia-Inducible Factor 1 (HIF-1), which regulates genes that enhance glycolysis and lactate production. In many hypoxic tumor regions, lactate serves as an alternative energy fuel, helping cancer cells survive glucose deprivation through conversion into pyruvate and participation in energy production [19,20]. Even in normoxic conditions, many tumors carry mutations that induce HIF-1 and keep these metabolic adaptations active [10]. A study by Schroeder et al. showed that after three days of a KD, the level of lactic acid was diminished in the tumor tissue of patients with head and neck cancer [21].

Additionally, the acidic pH of the TME, due to elevated lactate production, increases the permeability and helps the proliferation of cancer cells in surrounding tissues and additionally limits glucose availability for antitumor immune cells such as T lymphocytes and natural killer cells [19,22].

**Figure 2:**
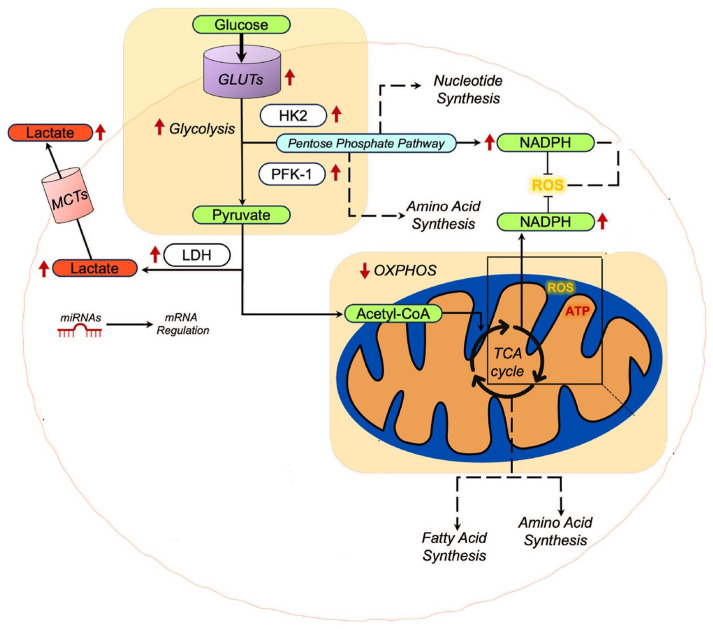
Metabolic reprogramming in cancer cells. Cancer cells, especially lung cancer, present an upregulation of glucose transporters (GLUTs) indicated by **red upward arrows**. This is linked with an increased reliance on glucose uptake and eventually on glycolysis to sustain tumor growth and survival. Pyruvate is transformed into lactate by **LDH**, which in turn exits tumor cells via monocarboxylate transporter 1 (MCT1). In this way, lactate can be used by oxygenated tumor cells, where it is converted into pyruvate, preserving glucose availability and sustaining energy production. Apart from energy production, lactate cycling is necessary for the survival of the tumor cells as it alters the acidity of the tumor microenvironment, stimulates angiogenesis, suppresses immune responses, and helps tumor invasion and metastasis. Another important step in cancer cell survival is the regeneration of NADPH, as it supports the biosynthesis of molecules such as fatty acids, nucleotides, and neurotransmitters, enhancing the survival of the cancer cells and additionally protecting them from oxidative stress. NADPH is crucial in maintaining redox homeostasis by acting as an antioxidant to inhibit surplus ROS from damaging the cell. Regeneration is achieved via the pentose phosphate pathway, using a part of the available glucose. As mentioned, in cancer cells, the mitochondrial OXPHOS is significantly reduced, partly due to alterations in mitochondrial oxidative metabolism, which involve functional and regulatory differences that decrease dependence on oxidative phosphorylation and allow other metabolic pathways, including the **TCA cycle** for biosynthetic precursors, to support tumor growth and survival. Red arrows indicate upregulation (up) or downregulation (down) of metabolic flux/expression; yellow T-bars indicate inhibition/neutralization of ROS; gray arrows represent standard metabolic pathways. HK2, hexokinase 2; PFK-1, phosphofructokinase-1; LDH, lactate dehydrogenase; NADPH, nicotinamide adenine dinucleotide phosphate (reduced form); TCA, tricarboxylic acid cycle. Adapted and modified from Mitaishvili et al., 2024, licensed under CC BY 4.0 [22]. It was modified by the authors to remove breast cancer–specific details while preserving general cancer metabolic mechanisms.

A schematic presentation of some of the alterations in the context of metabolic reprogramming of cancer cells is presented in Figure 2.

Together, these observations summarize the complexity of metabolic pathways in cancer, particularly in LC, and highlight the flexibility and adaptability of cancer cells that support survival and proliferation, which may limit the effectiveness of glucose restriction alone.

### 3.2. Current Guidelines and Recommendations

Nutrition is one of the basic aspects of supportive care in oncology, as it influences treatment tolerance, therapeutic efficacy, and overall quality of life [8,23]. Weight loss and nutritional disorders, including malnutrition and cancer cachexia, are common phenomena and are associated with multiple aspects of the disease. In this setting, there have been attempts to make recommendations for this group of patients [8].

The guidelines of the European Society for Clinical Nutrition and Metabolism (ESPEN) highlight that cancer patients should consume adequate protein (>1 g/kg/day and ideally 1.2–1.5 g/kg/day) as it enhances muscle anabolic activity and contributes to the treatment of cancer cachexia. As for the other components, there is no recommendation for an exact fat-to-carbohydrate ratio; however, the guidelines propose higher fat consumption and lower carbohydrates, especially in underweight and insulin-resistant cases. Additionally, while acknowledging the growing interest in specific dietary approaches, including preclinical data on the KD, ESPEN discourages restrictive diets that may compromise nutritional adequacy but supports prioritizing fat as an energy source within professionally supervised nutritional interventions [24].

Similarly, the European Society for Medical Oncology (ESMO) guidelines for the management of cancer cachexia recommend dietary patterns in which approximately half of non-protein calories derive from fats. Such patterns contribute to more effective coverage of basal energy metabolism, ensure high energy density with smaller food volumes, preserve protein for tissue repair, protect muscle mass from catabolic stimuli and reduce inflammation and insulin resistance [23].

The American Society of Clinical Oncology (ASCO) focuses on diet and physical activity during and after cancer treatment, without recommending a specific percentage of nutrients or supporting a specific dietary pattern [25]. 

Obesity seems to constitute a metabolic prognostic factor, as it is associated with an increased risk of developing various cancer types, reduced treatment response, and poorer clinical outcomes [26,27]. Metabolic disturbances accompanying obesity, such as insulin resistance, elevated levels of growth factors like IGF-1 and leptin, hyperinsulinemia, and chronic low-grade inflammation, contribute to the development of a pro-oncogenic environment by promoting cellular growth and adversely affecting treatment pharmacokinetics [28]. However, in LC, there are studies supporting the “paradoxical” beneficial effect of obesity on incidence, survival, and general outcomes [29]. This conclusion may arise due to the use of BMI as the sole parameter to define obesity, while other studies measuring central fat via imaging modalities have shown opposite effects of visceral obesity [30].

Accordingly, the ASCO guidelines do not recommend intentional weight loss during active cancer therapy, highlighting the need for further research and targeted metabolic interventions within oncologic care [25].

Specifically for LC, there have not been official nutritional guidelines or recommendations. An RCT that compared the existence or not of dietary counseling in LC patients showed better maintenance of body weight, higher energy and protein intake, and improved treatment response rates with counseling, although survival was not increased [31]. In a large retrospective case–control study of never smokers, Gorlova et al. identified that a “healthy eating” dietary pattern rich in fruits, vegetables, and low-fat foods was independently associated with a significantly reduced risk of LC [32]. The Mediterranean diet has been associated with a reduced risk of LC [33]. Additionally, certain micronutrients, including folate, vitamins B, C, D, and E isoforms, as well as selenium and phytochemicals, have been implicated in LC prevention through effects on DNA methylation, although data are limited and the need for supplementation remains unclear [34]. Interventions involving dietary counseling, high dietary protein, and n–3 fatty acid consumption show some benefits in a variety of outcomes in LC patients, which is in line with current nutrition guidelines [24,35].

### 3.3. Fundamentals of the Ketogenic Diet

The aim of KD is to change the primary energy source from glucose to ketones. By eliminating the consumption of carbohydrates and glucose, the body turns towards gluconeogenesis in the liver [36]. Under prolonged lack of glucose, glycogen in the liver and muscles is consumed, and the body enhances the FAO in the liver to provide energy. This results in increased production of ketone bodies (β-hydroxybutyrate (BHB) and acetoacetate (AcAc)), which are transported to peripheral tissues, enabling further energy production through mitochondrial oxidative processes [37]. They can be used as an energy source in all the vital organs except the red blood cells and the liver cells, as the former lack mitochondria and the latter the diaphorase enzymes. These organs derive energy in other ways: the red blood cells through glucose glycolysis and the liver through fatty acid oxidation [7].

Per acetyl-CoA unit entering the Krebs cycle, BHB yields more energy than glucose, making ketone bodies metabolically more efficient substrates [38]. By providing an oxygen-efficient alternative to fatty acids and a flexible complement to glucose, ketone bodies may satisfy energy needs during a caloric deficit and can limit oxidative injury mediated by ROS by increasing antioxidant signaling pathways [37,39]. So, beyond fuel, ketone bodies act as signaling molecules that modulate inflammation, oxidative stress, and gene expression, and, therefore, their role has been studied in many clinical conditions and diseases [40].

There are four main types of KD that primarily differ in macronutrient ratios. These include the classical KD (4:1 fat-to-carbohydrate-plus-protein ratio, corresponding to approximately 90% of total energy intake from fat); the medium-chain triglyceride (MCT) diet, which uses MCTs to enhance ketosis while allowing higher carbohydrate intake; the modified Atkins diet; and the low–glycemic index ketogenic diet, which permits slightly more carbohydrates, which are exclusively derived from low–glycemic index foods.

KD has been extensively studied in the context of drug-resistant epilepsy in children, and it is now included in the international treatment guidelines, as it has been shown to reduce seizure frequency and improve sleep patterns and behavior [40,41,42,43]. Beyond epilepsy, ketosis has shown neuroprotective effects in preclinical models of stroke, traumatic brain injury, spinal cord injury, and neurodegenerative diseases, with animal and preliminary human data also suggesting benefits in Alzheimer’s and Parkinson’s disease [40,44,45]. KD has also been investigated as a therapeutic strategy for obesity, type 2 diabetes, and other metabolic disorders, where meta-analyses support improvements in weight, glycemic control, and in several cardiometabolic risk markers, although LDL cholesterol often increases and long-term outcome data are limited [46,47]. In cardiovascular disease, including heart failure, the capacity of the heart to utilize fatty acids is diminished, and therefore, cardiomyocytes shift fuel use towards ketone bodies [40,48]. Enhanced myocardial ketone utilization appears adaptive and small trials of exogenous ketones or KD report improved hemodynamics and risk factors, but therapeutic ketosis is still considered an experimental adjunct rather than a guideline-recommended therapy [49].

**Figure 3:**
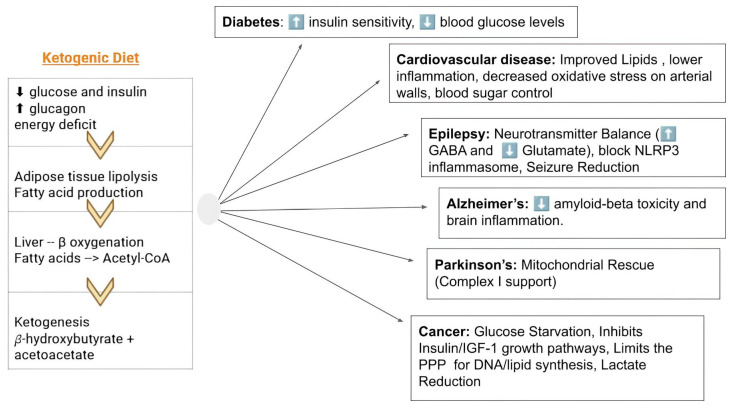
Therapeutic Mechanisms of the Ketogenic Diet across Multiple Pathologies. When glucose is absent, the body experiences a metabolic shift. The liver consumes its glycogen stores, and once depleted, a decrease in insulin levels triggers lipolysis, releasing fatty acids into the bloodstream. Fatty acids are then transported to the liver, where they are converted into ketones, which provide a highly efficient alternative energy source for the body. To provide energy for those cells that require glucose, such as red blood cells, the liver performs gluconeogenesis, creating necessary sugars from non-carbohydrate sources, such as glycerol and amino acids. This shift from a sugar-burning body to a fat-burning body helps preserve muscle mass while providing a constant energy source for vital organs. The figure also summarizes the multi-organ benefits of nutritional ketosis. By lowering systemic glucose and insulin, the ketogenic diet triggers a metabolic switch that disrupts the glycolytic pathways essential for tumor growth, provides an alternative energy substrate for insulin-resistant neurons, stabilizes neuronal excitability, and improves cardiovascular risk factors through enhanced fat oxidation and inflammatory regulation. PPP: Pentose phosphate pathway; GABA: γ-Aminobutyric acid; NLRP3: NOD-, LRR-, and pyrin domain–containing protein 3.

A summary of the metabolic alterations and the possible effect of KD in different pathologies is presented in Figure 3.

The safety of KD is well established due to the extensive experience in the field of refractory epilepsy in children. It is frequently associated with mild to moderate gastrointestinal symptoms, such as nausea and constipation. Micronutrient deficiencies, including inadequate intake of vitamins like B1, B3, B5, B7, and E, as well as minerals such as selenium, magnesium, and potassium, have also been reported when the diet is not properly designed [47,50]. Long-term and more serious complications may include fatty liver filtration, hypercalciuria, and possible nephrolithiasis [50]. Current evidence suggests that long-term adherence may be associated with unfavorable increases in LDL and total cholesterol as well as increased cardiovascular morbidity; however, available data are mixed and appear to additionally depend on the quality of dietary fat intake as well as individual patient characteristics [49,50,51]. Notably, clinical trials in oncology cohorts have demonstrated that while LDL may show a minimal increase, the overall metabolic profile often improves, specifically regarding the triglyceride-to-HDL ratio, with no out-of-reference values in other safety parameters [52].

Overall, outside its established role in refractory epilepsy and selected metabolic conditions, KD and therapeutic ketosis should be regarded as promising but investigational approaches that require further large, long-term, comparative clinical trials before routine implementation across other indications.

### 3.4. Ketogenic Diet in Cancer: Preclinical and Clinical Evidence in Different Cancer Types

Numerous preclinical studies investigated the potential role of KD in brain cancer, given its established use in drug-resistant epilepsy [41,42]. Studies in mice with malignant glioma have demonstrated that KD may reduce tumor growth and prolong survival [48,49]. As a standalone intervention, it was associated with modulation of oxidative stress and modification in gene expression patterns involved in the regulation of ROS levels, while in combination with brain radiotherapy, it enhanced the antitumor effects of radiation [53,54]. However, other preclinical studies showed that KD and more specifically BHB, did not protect the human glioma cell lines from cell death but prolonged survival of primary rat hippocampal neurons under glucose deprivation, suggesting that in some glioma models, KD’s antitumor effects may occur indirectly [55].

Panhans et al. reported a retrospective case series of 12 patients with central nervous system malignancies (CNS) who followed a supervised KD alongside standard oncologic care. Most patients achieved and maintained nutritional ketosis (*p* = 0.002), accompanied by moderate weight loss (5% of their total body weight, *p* = 0.005) and generally good tolerability, while several patients reported improvements in symptoms and selected cases showed radiographic signs of disease stability or reduced edema (42% of the cohort). The findings primarily support short-term safety and metabolic feasibility [56].

There are numerous case reports of CNS malignancies that explore the role of KD; however, clinical trial data remain limited, as most studies involve small participant numbers. In a single-arm study with only 20 patients with recurrent glioblastoma, the KD showed limited efficacy as a monotherapy but was associated with higher response rates and prolonged progression-free survival (PFS) when combined with antiangiogenesis therapies, such as bevacizumab (Objective Response Rate (ORR): 86%) [57]. Champ et al. retrospectively evaluated 6 patients with glioblastoma multiforme undergoing standard chemoradiotherapy followed by KD, showing that KD was safe, well tolerated, and capable of significantly reducing serum glucose levels even in the presence of corticosteroid therapy (*p* = 0.02), effectively reducing steroid-induced hyperglycemia, which is a known negative prognostic factor in glioblastoma. However, due to sample size, no conclusions regarding antitumor efficacy or survival benefit were drawn [12].

Similar studies published on this subject focused on different tumor types. A study conducted on 75 mice with prostate cancer showed that a no-carbo KD was associated with a significant reduction in tumor volumes (*p* = 0.009) and an increased survival rate compared with the classic Western diet (*p* = 0.006) [58]. This effect may be particularly relevant given that insulin is a potential growth factor for prostate cancer and that elevated insulin levels can promote tumor progression [59,60]. Consistently, in a randomized study in patients with recurrent prostate cancer, a trend toward a prolonged prostate-specific antigen (PSA) doubling time was seen in those following a KD, although that was not statistically significant (21.1 vs. 15.3 months) [56]. Although the primary intention-to-treat analysis did not reach statistical significance (*p* = 0.316), a post hoc analysis adjusting for weight-loss-induced hemoconcentration revealed a significantly slower PSA rise (*p* = 0.021), indicating that the diet may indeed influence tumor kinetics [61].

In breast cancer, factors like obesity, sarcopenia, insulin levels, and chronic hyperglycemia seem to negatively affect prognosis [62]. Randomized studies have shown that KD reduces BMI, body weight, and fat mass without muscle loss, improves metabolic markers like the triglyceride to HDL cholesterol ratio, reduces serum lactate (*p* = 0.02) and contributes to the reduction in insulin levels [62,63]. Additionally, in patients with locally advanced disease, it was linked with tumor size reduction and downstaging after 12 weeks of intervention [63].

Similarly, studies with the same design in rectal cancer showed that KD led to substantial weekly fat loss (*p* < 0.001) with preserved muscle and a trend toward better pathological tumor regression (pathological complete response: 25% (KD) vs. 12.5% (control)) [64].

Beyond the implementation of KD for its possible metabolic effects, there are data highlighting the synergistic effects of the diet with the standard oncological therapy, including chemotherapy, radiotherapy, and immunotherapy. KD and BHB activate AMP-activated protein kinase (AMPK) and act as endogenous histone deacetylase inhibitors (HDAC), leading to PD-L1 downregulation, enhanced type I interferon signaling, and upregulation of antigen-presentation genes in tumor and myeloid cells. In this way, it may potentiate CTLA-4 and PD-1/PD-L1 blockade [39,65]. In the same context, intermittent KD or BHB supplementation has been shown to convert immunologically “cold” tumors into more inflamed phenotypes, characterized by increased infiltration of cytotoxic CD8^+^ T cells and CXCR3-positive T cells, along with reduced regulatory T cells and myeloid-derived suppressor cells [65]. This shift has been associated with restored responsiveness to immune checkpoint inhibitors [65]. Other reviews have also identified KD and immunotherapy as a leading research frontier, but emphasize that clinical data are absent [66,67].

As mentioned above, by lowering glucose, insulin/IGF-1, and glycolytic flux while driving ketone-supported mitochondrial respiration, KD shifts redox balance toward higher NADH and ROS, selectively sensitizing tumors to DNA-damaging cytotoxics and radiation [10,14,17]. The possible synergistic effect of KD and radiation is based on these characteristics [68]. In pancreatic, glioma, head-and-neck, gastric and lung models, KD combined with chemotherapy or radiotherapy amplifies tumor growth delay and survival, partly via enhanced oxidative stress, ferroptosis, angiogenesis inhibition, and EMT reversal in the tumor microenvironment [16,18,21,53,54,55]

### 3.5. Preclinical and Clinical Evidence of Ketogenic Diet in Lung Cancer

Data on the possible impact of KD in lung malignancies are limited and mostly encompass preclinical studies.

A recently published study assessed different dietary patterns in a mouse model of LC and showed that ketogenic diets significantly reduced lung tumor burden compared with a Western diet, with the strongest protective effect observed when the KD was enriched with fish oil (*p* < 0.05). This superiority was linked to its ability to induce the highest ketone levels and promote a significant reduction in tumor-promoting prostaglandin E2 (*p* < 0.05), a known pro-inflammatory driver of tumor growth [69].

In mouse xenograft models bearing human lung cancer cell lines (NCI-H292 and A549), the effects of a KD were evaluated alone and in combination with radiotherapy and radio-chemotherapy. The study showed that KD combined with radiation or with carboplatin plus radiation slowed tumor growth compared with radiation alone (*p* < 0.05) and was associated with increased oxidative damage and inhibition of cancer cell proliferation [68].

In the same context, preliminary data from a conference abstract presented at the 2022 ASCO Annual Meeting suggested that in Lewis lung carcinoma xenografts, a calorie-restricted KD (70% of normal energy, KetoCal 4:1) increased blood ketones, slowed tumor growth (*p* < 0.001), and prolonged survival when combined with fractionated radiotherapy (*p* < 0.05), without major organ toxicity. These effects were further supported by a significant reduction in the proliferation marker Ki-67 (*p* < 0.05) [70].

An attempt to evaluate the possible role of ketone bodies in mice with cancer cachexia failed to show any benefit; on the contrary, simply restoring circulating ketones (via diet or supplements) did not show any improvement in cachexia or survival [71].

Despite data from preclinical findings, the main challenge in clinical studies remains patient adherence to the diet. A characteristic example is the only phase I study conducted to date by the University of Iowa, which assessed the tolerability and safety of a ketogenic diet in combination with chemotherapy and radiotherapy in patients with NSCLC. The results were discouraging, as only two of the seven enrolled patients were able to adhere to the diet and complete the study [72]. The limited number of participants and the even smaller number achieving and maintaining ketosis limit the evaluation of the possible effects of the diet and underline that it may be a difficult intervention to follow.

Two published case reports describe patients with advanced NSCLC who initiated a KD alongside or after standard oncologic treatment and experienced prolonged disease stabilization with acceptable tolerability. Evangeliou et al. reported a patient with metastatic NSCLC who maintained stable disease for 84 months by managing a Glucose-Ketone Index (GKI) between 1.0 and 2.0. Similarly, Tan et al. documented a patient with brain metastases who achieved a complete response in the brain and survived for at least 20 months after starting a calorie-restricted KD, despite having previously failed multiple lines of standard therapy; however, these remain low-level evidence [73,74].

Due to the limited availability of LC-specific studies, it is worth mentioning published evidence combining data from different solid tumors, including LC.

Apart from the metabolic changes, positron emission tomography/computed tomography (PET/CT) responses were evaluated in a case series of 37 patients with advanced cancer who additionally followed KD for 3 months. 6 participants had NSCLC. Although the study did not report the responses of the LC subgroup, one representative NSCLC case marked a significant reduction of 18F-FDG uptake alongside targeted therapy, suggesting comparatively favorable outcomes. Overall, the study concluded that KD was associated with PET-based metabolic responses and favorable survival estimates. Patients exhibiting a favorable profile of higher albumin and lower glucose and CRP levels after three months of ketogenic intervention demonstrated a significantly improved 1-year survival rate of 100% compared to 45.4% (*p* < 0.001). Furthermore, this metabolic shift was associated with an 84% reduction in the risk of death (HR = 0.16; *p* = 0.003) [75].

In contrast to this exploratory case series, another study with a similar heterogeneous composition of stage II and III solid tumors, including LC, applied a randomized controlled design to evaluate the quality of life and the mental status in patients following a KD enriched with MCT for four months compared with a control group receiving a standard diet. The intervention group had an improvement in their self-reported quality of life (QoL) over time, as well as an improvement in their mental health as compared to those in the control group (*p* = 0.005). Most participants (82.1%) maintained ketosis even later in the course of the disease. Although the observed positive effects may partly reflect a sense of active patient participation in therapy, a positive association was also reported between patient health scores and urinary ketone levels (r = 0.547, *p* = 0.002), suggesting that the deeper the ketosis, the better the patients’ overall QoL [76].

A retrospective Stage IV NSCLC cohort treated with chemotherapy supported by several metabolic interventions, such as KD, hyperthermia, and hyperbaric oxygen therapy, in order to simultaneously target multiple metabolic pathways, reported high ORR (86.4%, with 18.2% of patients achieving a complete response) and a median OS of ≈43 months, but with no control arm and strong selection bias concerns [77].

At present, a clinical trial from the University of Birmingham is ongoing, assessing the addition of continuous ketogenic diet therapy to standard chemotherapy and immunotherapy in patients with advanced squamous cell LC, with completion expected in January 2027 [78].

### 3.6. Clinical Considerations in Lung Cancer: Limitations, Ethics, and Future Perspectives

Despite enduring data on shifts in cancer metabolism and the potential effect of ketogenic metabolic therapy, most of the clinical data mentioned in this review come from other solid tumors, particularly glioblastoma, breast, and rectal cancers. Currently, lung cancer–related evidence is limited to preclinical studies and a few phase I feasibility studies and case reports. Thus, the clinical application of KD in LC remains investigational, and findings from other malignancies should be extrapolated cautiously. This paucity of evidence constitutes a clear limitation of the review and should be considered when interpreting its applicability in LC patients. Furthermore, most of the available literature is based on preclinical trials. This data must be used with caution, as differences in tumor biology, metabolic flexibility, and experimental models limit their direct translation into clinical practice.

The safety of KD is well-established due to its extensive use in children with drug-resistant epilepsy, as it is part of the disease’s international guidelines [42]. However, its application in the oncological setting, and especially in LC cases, constitutes a personal, supplementary initiative with possible positive effects, although not officially recommended by international scientific societies. For this reason, the application requires strict context with a multidisciplinary team, including nutritionists with expertise in the field, along with the oncologists and the rest of the medical team. Even though the adverse events of KD are frequently mild to moderate, multidisciplinary supervision reduces their incidence [51].

Regular monitoring of the basic electrolytes, the lipid profile, and the renal function, along with vitamin levels, is necessary to avoid side effects and detect deficiencies [47,50]. Supplementation of electrolytes and vitamins is common when KD is administered [47,50]. A study in a pediatric population with epilepsy demonstrated that KDs are frequently associated with micronutrient deficiencies, sometimes persisting even under supplementation [79]. Another study assessing the micronutrients in adults under KD showed an incompatibility between the intake of micronutrients and their serum levels, which were within reference ranges during a short period of ketosis; however, serum calcium and water-soluble vitamin levels may decrease over time [79,80]. Therefore, it is necessary to monitor the plasma availability of the most common micronutrients and follow careful food selection and individualized supplementation protocols, depending on each patient’s needs and clinical status. Additionally, blood or urine glucose and ketone bodies should be monitored weekly to control the level of ketosis and modify the diet to maintain the possible therapeutic effect.

A significant concern regarding the clinical application of the KD in oncology is its feasibility and tolerability in patients whose nutritional status is already compromised by the malignancy itself. Generally, approximately 40% of LC patients experience malnutrition [81]. It is worth mentioning the differences between malnutrition and cachexia to evaluate the possible effect of KD on these situations. While malnutrition is defined by diminished intake or absorption of nutrients, resulting in the loss of lean body mass, cachexia is a more complicated state, driven by tumor-secreted catabolic factors causing anorexia, weight loss with emphasis on muscle wasting, and elevated pro-inflammatory cytokines that promote insulin resistance [82]. It also involves hormonal and metabolic disturbances such as increased gluconeogenesis, suppressed protein synthesis, and enhanced lipolysis [82]. Research on KD in cancer-related cachexia and sarcopenia shows mixed effects. In murine models of IL-6-producing cancers, KD delayed tumor growth but paradoxically accelerated cachexia onset and shortened survival. This effect was linked to corticosterone deficiency that worsened metabolic stress; glucocorticoid treatment mitigated these negative effects while preserving tumor suppression [18]. In LC mouse models, restoration of circulating ketone bodies through a KD or ketone supplementation did not improve body weight maintenance or survival, despite successfully increasing ketosis [71]. These findings suggest that enhancing ketone availability and hepatic PPARα activity (a key regulator of fatty acid oxidation and ketone metabolism) does not alter cachexia progression, highlighting the possible limited impact of KD on the underlying mechanisms of cancer cachexia [71]. For pancreatic cancer-associated cachexia, KD combined with chemotherapy showed promise in preserving skeletal muscle mass and strength in mice, suggesting potential benefits against sarcopenia through mechanisms like autophagy inhibition [83,84]. KD in cancer patients has demonstrated the ability to reduce body weight primarily through fat loss while generally preserving muscle mass. Clinical trials mentioned above, including phase I studies in breast and rectal cancer patients undergoing radiotherapy, showed that KD led to significant reductions in fat mass without detrimental effects on skeletal muscle or fat-free mass, indicating muscle preservation during treatment [62,63,64]. Panhans et al., in CNS malignancies, also support the feasibility of KD with maintained nutrient adequacy and muscle preservation [56]. Meta-analyses confirm that KD safely reduces body weight and fat mass in cancer patients but highlight the importance of individualized nutritional assessment to avoid exacerbating malnutrition or muscle loss [51,85]. It appears that the diet’s high-fat, moderate-protein composition supports adequate nutrient intake and energy needs, helping maintain nutritional adequacy despite weight loss [86]. However, while KD may influence tumor metabolism and systemic factors related to cachexia, its impact on nutritional status in cancer patients remains inconclusive. Consequently, the ethical implementation of a KD in advanced LC requires a highly individualized nutritional assessment and support where the preservation of muscle mass and quality of life is prioritized over strict adherence to ketosis, particularly in patients at high risk for refractory cachexia.

In the same context, considering that the adoption of specific dietary interventions is not part of the current international guidelines, every attempt outside of the controlled environment of a clinical trial should be done with extreme caution, especially in consideration of the potential for treatment interference and nutritional depletion. To mitigate these risks, the implementation of a KD in lung cancer must occur within a multidisciplinary framework, ensuring that oncological treatment remains the priority and that dietary changes are professionally supervised by specialized dietitians. Appropriate monitoring strategies, as mentioned above, are essential to achieve ketosis while minimizing adverse effects. Unsupervised adoption of the KD by patients may lead to nutritional inadequacy and adverse events that can compromise the tolerance of standard oncological therapies and lead to excessive weight loss.

Another challenge in the implementation of KD is adherence to the diet, as systematic reviews of cancer patients report that only about half of patients can maintain the diet for the intended intervention period, with completion rates as low as 49% across studies [56,62,63,64,87]. Most of the available trials were short (often <6 months), and, as a result, long-term KD adherence is uncertain [51]. The KETOCOMP trials describe KDs as feasible during radiotherapy; sustained ketosis and progressive fat-mass loss indicate reasonably good compliance over the RT period, but follow-up beyond that window is lacking [62,64]. Multiple, interacting factors influence compliance and practical implementation. The restrictiveness and palatability of low-carbohydrate, high-fat diets make long-term use difficult, especially when integrating the diet into family eating patterns and social situations; patients often consider the regimen burdensome over time [87]. Early gastrointestinal side effects such as constipation, diarrhea, and fatigue—partly related to low fiber intake and rapid fat escalation—can further undermine adherence, particularly in the first 4 weeks before adaptation [87]. Motivation and perceived feasibility are also critical: patients who are highly motivated and have strong caregiver support, as in some glioblastoma trials and case series, achieve high rates of nutritional ketosis and sustained adherence [56,62,64]. Practical skills and attitudes also matter in an online survey, patients who enjoyed cooking could readily identify suitable recipes, and liked fatty foods were much more likely to follow the diet [88]. Support from dietitians experienced in KD, provision of ketogenic products and recipes or prepared meals, and close monitoring with ketone measurements can substantially improve feasibility and adherence in clinical settings [56]. Conversely, lack of professional counseling, limited institutional KD services, cost of specialized foods, and clinicians’ uncertainty or hesitancy about KD all represent additional barriers to implementation [89,90].

As for future perspectives, there are some limited preclinical data suggesting a more tailored approach based on metabolic and molecular subtyping when using KD as supportive therapy in cancer patients; however, there are no large prospective trials yet applying these subtypes to guide ketogenic therapy. Additionally, most of the trials related to the topic do not stratify patients by metabolic phenotype, which limits the translation of these subtyping frameworks into practice. Large pan-cancer bioinformatic analyses integrating glycolysis and ketone-body metabolism genes across >10,000 tumors have defined distinct metabolic subtypes, showing that tumors with high glycolysis but low ketone-body utilization are more likely to benefit from ketogenic dietary therapy [91]. Similar results were derived from a study in colon cancer [92]. In LC models, tumor cells can switch from glucose to ketone-based metabolism under glucose limitation; a KD can paradoxically support their growth but simultaneously exposes new vulnerabilities to inhibitors of the ketone transporter MCT1 and fatty-acid synthase, illustrating how KT might be combined with targeted drugs rather than used alone [93]. A comprehensive LC metabolism review states that oncogenic drivers imprint distinct metabolic states and argues that genotype-plus-metabolic profiling will be needed to deploy metabolism-targeted strategies safely, including dietary ones [94]. Additionally, existing data show metabolic plasticity under KD and identify candidate dependencies (MCT1/FASN, AMPK, and oxidative stress pathways) [68,70,93]. These approaches may, in the future, help guide personalized use of ketogenic therapy by matching patients to specific metabolic profiles, while, for now, this personalization is still almost entirely preclinical or computational.

## 4. Conclusions

Available data in oncology also suggest that, when combined with intensive systemic therapies such as chemotherapy and chemoradiation, KD generally does not increase treatment-related toxicity or worsen liver/kidney function in most early-phase and randomized trials across solid tumors. However, feasibility and adherence vary widely, with dietary burden representing a major challenge, particularly given the compromised physiological state of patients with LC. Therefore, in the context of limited lung-specific efficacy data and adherence concerns, any use of KD in lung cancer should remain experimental, closely supervised, and integrated within standard oncologic and nutritional care.

## Data Availability

No new data were created or analyzed in this study. Data sharing is not applicable to this article.

## Abbreviations

The following abbreviations are used in this manuscript:

AcAc	Acetoacetate
AMPK	AMP-activated protein kinase
ASCO	American Society of Clinical Oncology
ATP	Adenosine triphosphate
BHB	β-hydroxybutyrate
BMI	Body mass index
CNS	Central nervous system
CXCR3	C-X-C motif chemokine receptor 3
ESMO	European Society for Medical Oncology
ESPEN	European Society for Clinical Nutrition and Metabolism
FAO	Fatty acid oxidation
GKI	Glucose-Ketone Index
GLUT1	Glucose transporter 1
HDAC	histone deacetylase inhibitors
HIF-1	Hypoxia-Inducible Factor 1
IGF-1	Insulin-like growth factor-1
QoL	Quality of Life
KD	Ketogenic diet
LC	Lung cancer
MCT	Medium-chain triglyceride
MCT1	monocarboxylate transporter 1
NADH	Nicotinamide adenine dinucleotide
NADPH	Nicotinamide adenine dinucleotide phosphate
NSCLC	Non-small cell lung cancer
ORR	Objective response rate
OXPHOS	Oxidative phosphorylation
PPARα	Peroxisome proliferator-activated receptor alpha
PET/CT	Positron Emission Tomography/Computed Tomography
PFS	Progression-free survival
PPP	Pentose phosphate pathway
PSA	prostate-specific antigen
ROS	Reactive oxygen species
TCA	Tricarboxylic acid cycle
TME	Tumor microenvironment
